# Zinc-α2-glycoprotein 1 promotes EMT in colorectal cancer by filamin A mediated focal adhesion pathway

**DOI:** 10.7150/jca.35380

**Published:** 2019-08-29

**Authors:** Meiling Ji, Wenxiang Li, Guodong He, Dexiang Zhu, Shixu Lv, Wentao Tang, Mi Jian, Peng Zheng, Liangliang Yang, Zhipeng Qi, Yihao Mao, Li Ren, Yunshi Zhong, Yongjiu Tu, Ye Wei, Jianmin Xu

**Affiliations:** 1 Department of General Surgery, Zhongshan Hospital Fudan University, Shanghai, China; 2 Department of Surgical Oncology, First Affiliated Hospital of Wenzhou Medical University, Wenzhou, China; 3 Departmentof Endoscopic Center, Zhongshan Hospital Fudan University, Shanghai, China; 4 Surgical Department, Hospital 174 of PLA, Xiamen, Fujian, China

**Keywords:** AZGP1, colorectal cancer, FLNA, metastasis, adhesion pathway

## Abstract

Liver metastasis is the main reason for the poor prognosis of colorectal cancer, and identifying molecules involved in liver metastases of colorectal cancer may provide effective therapeutic targets. Zinc-α2-glycoprotein 1(AZGP1) is a candidate biomarker for diagnosis and prognosis in cancer. However, its function and molecular mechanism in metastatic colorectal cancer remains largely unknown. We previously found that up-regulated AZGP1 promotes proliferation, migration and invasion in colorectal cancer cell line, here we elucidated the mechanism of AZGP1 in regulating metastasis. In this article, we found that AZGP1 was also highly expressed in colorectal cancer tissues with liver metastasis relative to those without metastasis, and abundant expression of AZGP1 was associated with poor prognosis, also, AZGP1 down regulation prevented cell metastasis in vivo and in vitro. We further demonstrated that AZGP1 promotes metastasis by regulating the epithelial-mesenchymal transition (EMT) and associating with molecules involved in the focal adhesion pathway, including the adhesion molecule FLNA, which acts as an important protein interactor. More importantly, AZGP1 down regulation inhibited the phosphorylation of FLNA mediated by the restrain of PAK2 kinase, thereby inducing its proteolysis and subsequently affecting its subcellular localization, where it regulates the EMT and promotes metastasis. Collectively, these results highlight AZGP1 as a new and promising therapeutic molecule for liver metastatic colorectal cancer.

## Introduction

Colorectal cancer (CRC) is one of the top five most frequent malignant tumors worldwide. Despite many advances in the diagnosis and therapeutic improvements, the prognosis of CRC patients remains poor. The most important factor is high frequency of metastasis and recurrence 1. Therefore to search potential biomarkers for liver metastasis predication and elucidate the exact molecular mechanisms may provide possibilities for improving patient outcomes.

Zinc-a2-glycoprotein (AZGP1, ZAG) is a secreted41-kDa protein that was initially identified and purified in human serum 2. AZGP1 is involved in lipid metabolism as an adipokine with an MHC class I-like protein fold and is functionally implicated in cell cycle 3-6. AZGP1 was also identified as an important factor in regulating tumor carcinogenesis, including lung, prostate, breast, liver and gastrointestinal tumor 7-12. On the one hand, its abnormal expression can be used as an important indicator for prognosis. In prostate cancer, the loss of AZGP1 is associated with worse clinical outcomes and a risk of recurrence, independently forecasts biochemical relapse 13-15. Decreased expression of AZGP1 is associated with a poor prognosis in primary gastric cancer and hepatocellular carcinoma 9,10. Also, AZGP1 was identified as a potential predictive biomarker for cancer and can be used for early diagnosis. In some cancers, AZGP1 shows significant diagnostic value as a serum marker. In prostate cancer, elevated serum ZAG (AZGP1) levels may occur early in progression before it can be detected by a digital rectal exam or elevated PSA 16. A previous study enrolled two independent cohorts of 868 individuals with CRC and indicated that an increased level of AZGP1 in the serum was significantly associated with a poorer overall survival (OS) and disease-free survival (DFS), indicating it as a reliable serum prognostic biomarker for CRC 17. Additionally, the combination of AZGP1 with the traditional serum biomarkers CEA and CA19-9 could result in better diagnostic results in colon cancer 11. All of these showed a huge clinical perspective. We previously demonstrated that over-expressed AZGP1 promoted proliferation and metastasis in HCT116 cell^18^, and to further explore the mechanism of metastasis in colorectal cancer deserves great significance.

Epithelial-to-mesenchymal trans-differentiation (EMT), which often leads to aggressive cancer progression, is a crucial event in the carcinogenesis of CRC. EMT is characterized by a loss of epithelial characteristics and the acquisition of a mesenchymal phenotype. AZGP1 was also shown to regulate metastasis. The analysis of AZGP1 expression in malignant prostate epithelium in prostatectomy specimens from 228 prostate cancer patients revealed that absent or weak AZGP1 expression was associated with clinical recurrence, including localized recurrence, metastasis, or death. These results suggest that AZGP1 may be an accurate and timely indicator of the risk of metastatic progression 19. AZGP1 has also been determined to play an important role in the TGF-β1-induced epithelial-to-mesenchymal transition (EMT) in pancreatic cancer and hepatocellular carcinoma. Silencing AZGP1 dramatically increased invasiveness and induced a mesenchymal phenotype 20, 21. However, in CRC, the mechanism by which AZGP1 regulates metastasis and its therapeutic potential remain largely unknown.

In this study, we found that AZGP1 expression was significantly increased in tissues from patients with liver metastasis relative to those without distant metastasis. Additionally, increased expression of AZGP1 indicated a poorer prognosis. Most importantly, our results demonstrated that AZGP1 silencing blocks the EMT and reduces metastasis in vivo and in vitro, which may be mediated by filamin A (FLNA) and the focal adhesion pathway.

## Materials and Methods

### Patient population

A total of 280 CRC patients from Zhongshan Hospital of Fudan University (Shanghai, China) were enrolled between January 2001 and March 2010. Clinicopathologic data were retrieved from our prospectively constructed CRC database. Tumor stage was determined according to the 7^th^ edition of the International Union Against Cancer (UICC)/American Joint Committee on Cancer (AJCC) TNM classification. Routine chemoradiotherapy had been given postoperatively to patients with advanced-stage disease. Ethical approval was obtained from the Clinical Research Ethics Committee of Zhongshan Hospital of Fudan University. Signed informed consent was obtained from all patients for the acquisition and use of patient tissue samples and anonymized clinical data. The follow-up data were regularly acquired through outpatient visits, telephone calls, or office visits. The median follow-up time was 56.5 months (range 1-144 months). OS was calculated from the day of surgery to the date of death due to CRC or the date of the last follow-up.

### Cell lines and culture

Human HCT116 and SW480 CRC cell lines and human embryonic kidney 293 cells were obtained from the cell bank of the Chinese Academy of Sciences (Shanghai, China). All cell lines were routinely maintained in DMEM (Gibco, USA) supplemented with 10% fetal bovine serum and 1% penicillin/streptomycin at 37 °C in a humidified chamber under 5% CO_2_.

### Immunohistochemistry

Formalin-fixed, paraffin-embedded sections were stained by a non-biotin enzymatic method (DAKO, Japan) for the specific detection of AZGP1 expression (Sigma, USA) in CRC tissues. The immunohistochemistry (IHC) results were calculated as the product of two independent scores: the proportion of positive tumor cells in the tissues (0=0%, 1=1%-10%, 2=11%-50%, 3=51%-80%, and 4=81-100%) and the average intensity of positive tumor cells in the tumor tissues (no staining=0, weak staining=1, moderate staining=2, and strong staining=3). The final score was determined by multiplying the staining intensity score with that of the positive proportion. Scores were determined independently by two senior pathologists. The final score was classified as low- or high-expression using the median value as the cut-off.

### Transwell assay

Cells were trypsinized, re-suspended in low serum media (0.1% FBS), and seeded at a density of 1 × 10^4^ with 100 µl per well. Six hundred microliters of media containing 10% FBS were added to the lower chamber (8 μm pore size, BD Biosciences, USA). After 48 h, the chambers were washed twice with PBS, and the cells on the apical side of each insert were scraped off. The invaded cells were fixed with 4% paraformaldehyde and stained with crystal violet. Cells were counted under a microscope.

### Immunofluorescence analysis

Cells were cultured on coverslips and fixed with 4% paraformaldehyde for 15 min, followed by permeabilization with ice-cold 95% methanol for 30 min. Cells were washed with PBS, blocked with 3% BSA/PBS/0.1% Triton-X for 1 h at room temperature and incubated with the appropriate primary antibody. After overnight incubation at 4°C, cells were washed, incubated with the fluorescence-labeled secondary antibody for 1 h at room temperature and mounted with anti-fade medium including DAPI. Images were captured by fluorescence microscopy (Olympus fv1200, Japan). The gain and offset settings were fixed across all samples.

### Mouse model of liver metastasis

Liver metastasis assays were performed using an intra-splendid injection CRC mouse model as previously described 22, 23. HCT116 cells (1×10^4^) were injected into the spleens of BALB/c mice. Animals were weighed regularly before the experimental endpoint. Mice were sacrificed 39 days later, and the rate of tumor formation and surface liver nodules were counted. All animal experiments were performed in accordance with NIH guidelines for the use of experimental animals.

### Immunoprecipitation

Cells were lysed in 1ml of chilled RIPA buffer, and the cleared lysate (400 µl) was incubated with 5 µl of the indicated antibody (AZGP1, Sigma) and 20 µl of protein-A agarose beads (Repligen, USA). The reaction was brought up to a final volume of 500µl with IP buffer. After constant rotation for 3 hours, beads were resuspended in 2× SDS buffer and subjected to Western blot analysis.

### Statistical analysis

Results are expressed as the mean ± SEM of at least 3 independent experiments. A paired Student's t-test was performed for data analysis. All statistical analyses were performed using GraphPad Prism software version 5.02 (GraphPad Software, Inc.).

## Results

### AZGP1 is highly expressed in CRC tissues with liver metastasis

**Figure 1:**
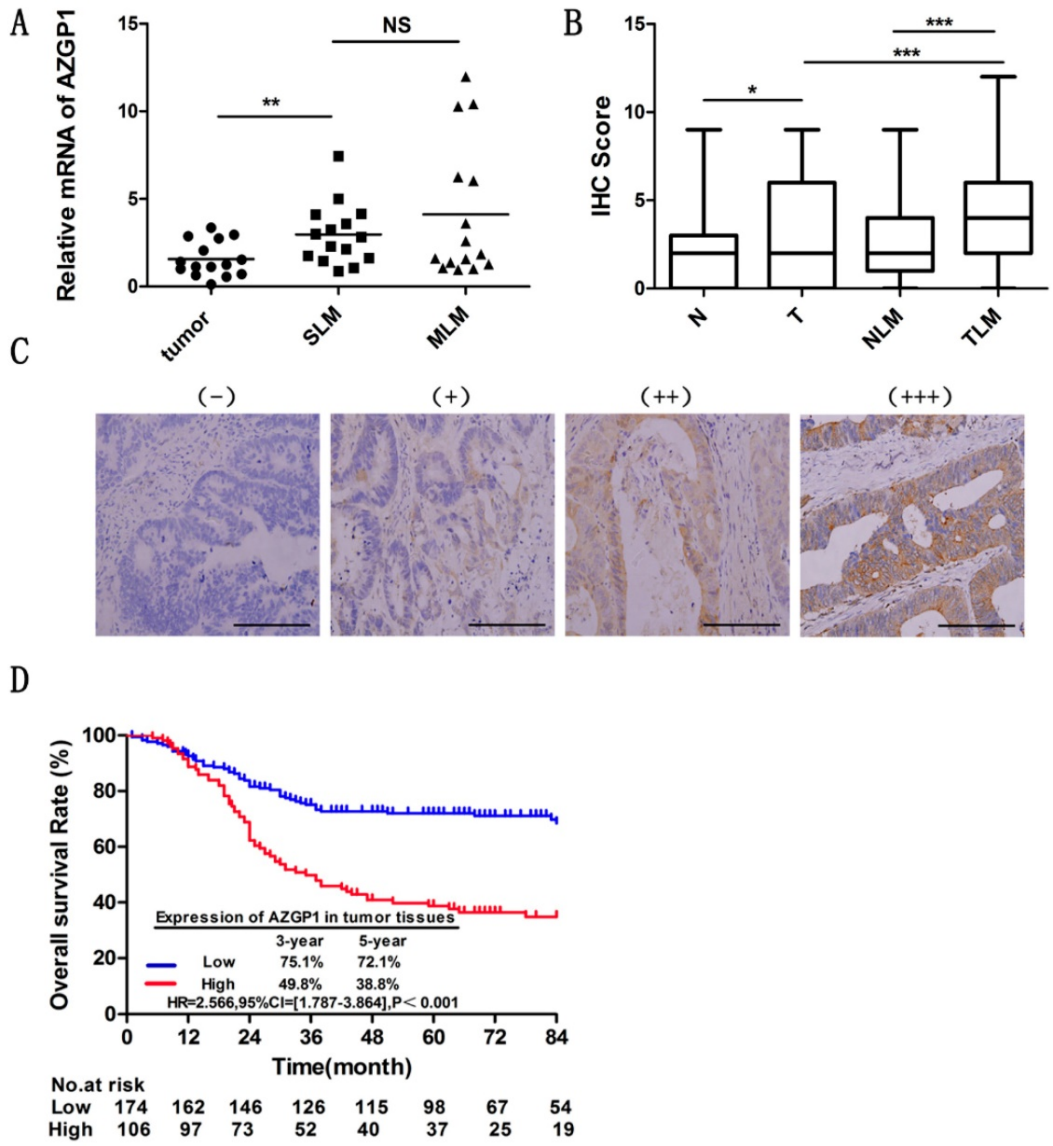
** AZGP1 is highly expressed in colorectal cancer tissues with liver metastasis, and high AZGP1expression in tumor tissues indicates a worse prognosis.** (A) mRNA expression of AZGP1 was verified by qPCR(n=15,P=0.0034).SLM: Simultaneous liver metastases, MLM: Metachronous liver metastases. (B) Protein expression of AZGP1 was examined by IHC. AZGP1 IHC staining score was higher in tumor tissues than in normal tissues, besides, it was significantly higher in subjects with liver metastasis than those without. N: normal; T: tumor; NLM: normal with liver metastasis; TLM: tumor with liver metastasis (C) Representative images of IHC staining intensity: (-) no staining; (+) weak staining; (++) moderate staining; (+++) strong staining. Scale bar: 25µm. (D) Kaplan-Meier survival curve of AZGP1 expression in tumor tissues from CRC patients after primary tumor resection. HR: hazard ratio; CI: confidence interval; P value: log-rank test. *P<0.05, **P<0.01, ***P<0.001.

Liver is the most frequent site of metastases from CRC. To figure out the liver metastasis of colorectal cancer, we obtained abnormal expressed genes through 6 patients without liver metastasis and 5 patients with liver metastasis by the HG-U133 microarray (Affymetrix), among these, AZGP1 was one of the most significant highly expressed molecules. To explore the role of AZGP1 in liver metastastic colorectal cancer, firstly, we verified the mRNA expression by qPCR, the result showed that AZGP1 was more abundant in primary tumors with liver metastasis than those without liver metastasis, besides, in metachronous metastasis primary tumors, it was higher than simultaneous metastasis tumor, although there was no statistical difference (Fig. 1A). To verify these results, the protein expression of AZGP1 was examined by IHC in a larger sample size containing 181 patients without liver metastasis and 99 with liver metastasis. Representative images show four different AZGP1 expression patterns (Fig. 1C). The IHC scores assay certificated AZGP1 were especially higher in subjects with liver metastasis than those without (Fig. 1B).

### AZGP1 expression in tumor tissues is associated with clinic-pathological characteristics

For IHC score, the median value was defined as the cut-off for the definition of AZGP1 high- and low-expression subgroups. As showed in Table 1, a total of 106 samples exhibited high expression, and 174 samples manifested low expression. High AZGP1 expression was associated with clinical stage and histological type. Patients with high AZGP1 expression were more frequently in stage IV than stage I-III (P=0.003). The intensity of AZGP1 in mucinous adenocarcinoma was significantly higher than in non-mucinous adenocarcinoma (P=0.029). No association was observed in other clinic-pathological characteristics (Table 1).

### High AZGP1 expression in tumor tissues indicates a worse prognosis of CRC

To investigate the effect of AZGP1 expression on the prognosis of CRC patients with liver metastasis, the Kaplan-Meier method was conducted. The median follow-up time for all patients was 56.50 months. The OS of patients with high AZGP1 expression was significantly worse than that of patients with low expression (HR=2.566, 95% CI=[1.787-3.864], P<0.001). For patients with high AZGP1 expression, the 3- and 5-year OS rates were 49.8% and 38.8%, respectively. For patients with low AZGP1 expression, the 3- and 5-year OS rates were 75.1% and 72.1%, respectively (Fig. 1D).

### Silencing AZGP1 prevents CRC metastasis by inhibiting the EMT in vitro and in vivo

**Figure 2:**
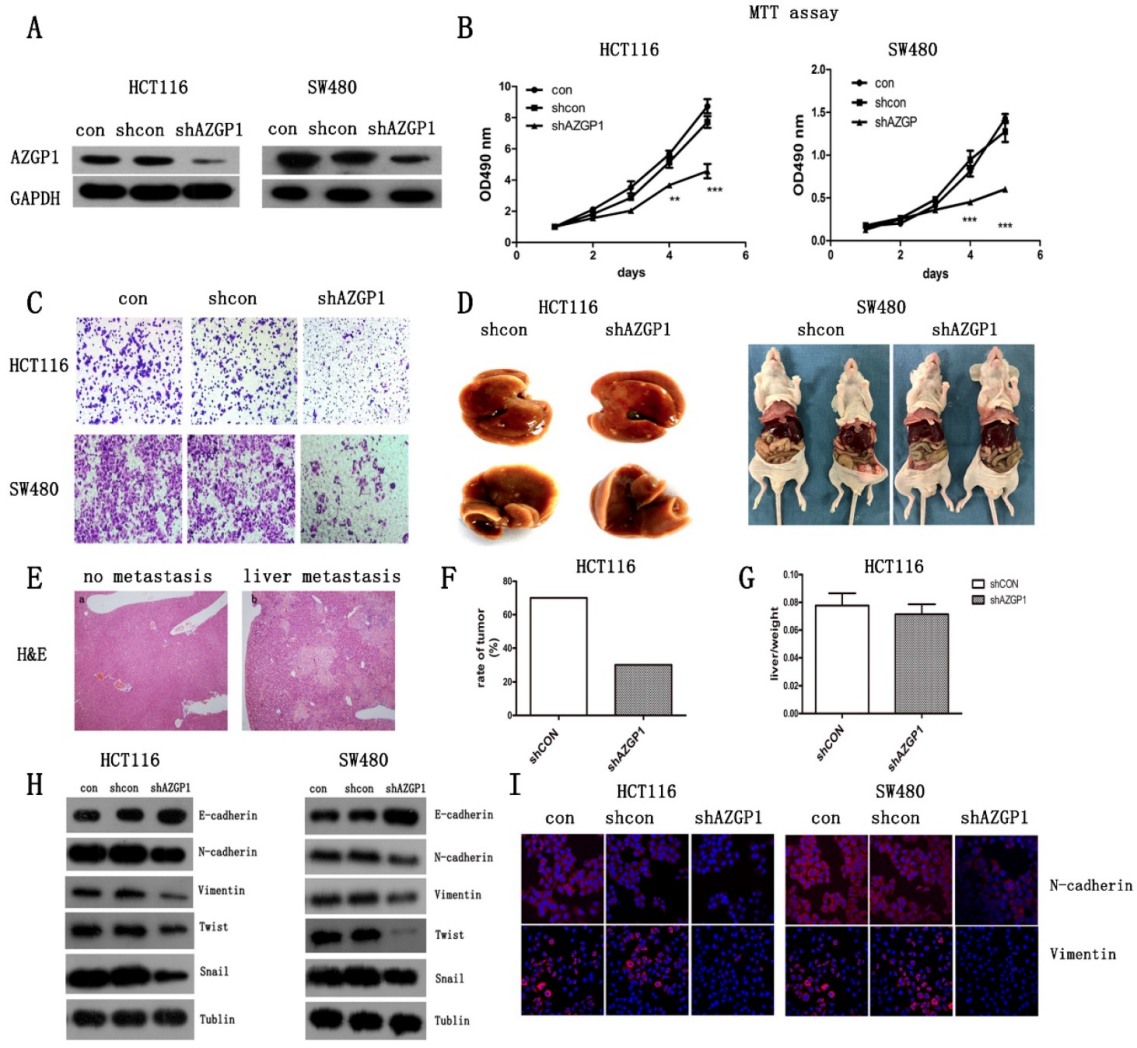
** Silencing AZGP1 prevents colorectal cancer metastasis by inhibiting the EMT in vitro and in vivo.** (A) Western Blot assay the efficiency of lentivirus of shRNA at AZGP1. (B) MTT assay the proliferation of colorectal cancer cell when silencing AZGP1. (C) Transwell migration assay was performed on HCT116 and SW480 cells when silencing AZGP1. Representative images are shown. (D-G) A liver metastasis model was used to examine metastasis in vivo. The weights of mice were monitored during the mold. After the appearance of cachexia, mice were sacrificed, representative liver with metastasis (D), representative H&E staining of a liver with nodules, liver metastases ratio (F) and the liver to body weight ratio (G) were determined. (H) Western blot analysis of EMT markers. (I) Representative immunofluorescence images of EMT markers N-cadherin and Vimentin. Short hairpin RNA (shRNA) targeting the human AZGP1 gene was designed as follows: sense 5'-TGGTTGTGAGATCGAGAATAA-3'.

Abnormally high expression of AZGP1 in CRC tissues with liver metastasis indicated that it correlated with cancer metastasis. To explore its exact role in cancer metastasis, CRC cell lines HCT116 and SW480 were infected with the lentivirus containing either shRNA targeting AZGP1 (shAZGP1) or a control scrambled shRNA (shCON), the efficiency of AZGP1 knockdown was verified by Western Blot (Fig. 2A). AZGP1 downregulation significantly impaired the proliferation of HCT116 and SW480 cells by MTT assay after 4 days (Fig. 2B) and the migration by transwell assays 48h later (Fig. 2C). Moreover, the establishment of a liver metastatic cancer model of CRC by injection into the spleen envelope caused a significant decrease in the rate of liver metastasis (Fig. 2F), stably transfected shRNA or shAZGP1 HCT116 cell lines were used for in vivo assays. Each group had ten mice, after seeding the cells, mice were monitored every 3 days for weight. Thirty-nine days later, 2 of them died in control group and 1 died in knockdown group, the remaining mice were sacrificed, 5 mice showed macroscopic metastasis in the liver of control group while 2 mice developed CRLM in knockdown group. The representative livers are shown in Figure 2D. However, the liver to body weight ratio was not affected (Fig. 2G). The liver node was stained by H&E (Fig. 2E). All of these showed that AZGP1 downregulated group generated less tumor foci. Therefore, AZGP1 is an important regulatory molecule for CRC metastasis not only in vitro but also in vivo. To further explore the induction of migration, we determined whether AZGP1 regulates epithelial and mesenchymal adhesion molecules. Lentivirus-mediated downregulation of AZGP1 increased the protein levels of the epithelial marker E-cadherin and simultaneously decreased those of various mesenchymal markers, such as N-cadherin, Twist1, Snail and Vimentin (Fig. 2H). These results were consistent with the immunofluorescence staining of EMT markers N-cadherin and Vimentin (Fig. 2I). These data support the role for AZGP1 in regulating the EMT transition. Overall, AZGP1 downregulation impairs the migration of CRC cells and restrains the EMT.

### AZGP1 promotes the EMT by interacting with FLNA

**Figure 3:**
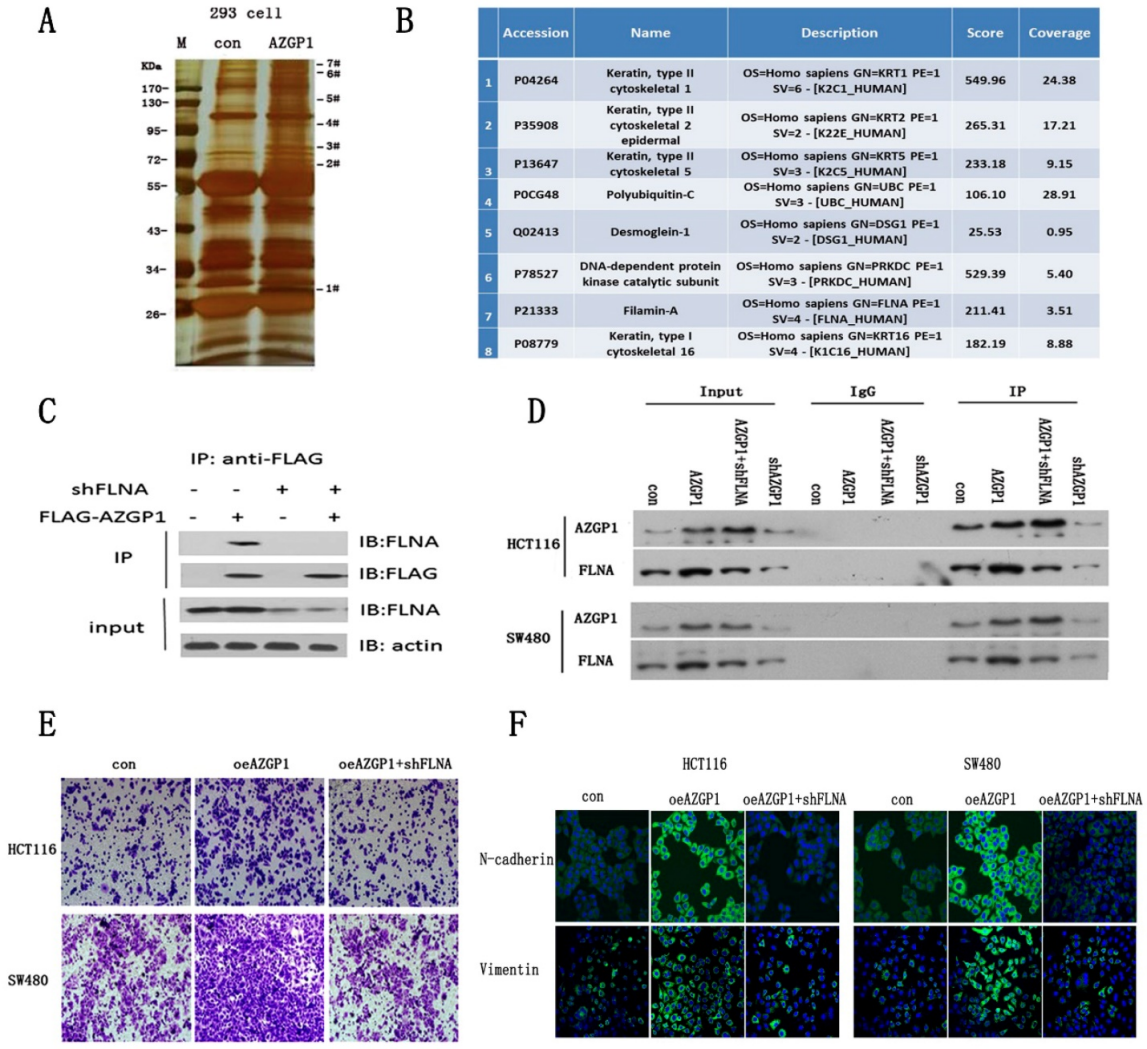
** AZGP1 promotes the EMT by interacting with FLNA.** (A) An immunoprecipitation assay was conducted to identify proteins that bind to AZGP1.The gel was silver stained. (B)Candidate binding proteins from Mass scan analysis.(C)Co-immunoprecipitation of AZGP1 and FLNA was conducted in 293 cells.(D)The interaction between FLNA and AZGP1 was confirmed in HCT116 and SW480 cells.(E)Rescue assay of migration by transwell was performed when overexpressing AZGP1 and knocking down FLNA. (F)Immunofluorescence staining of EMT markers in overexpressing AZGP1 (oeAZGP1) and knocking down FLNA.

To further investigate the molecular mechanism by which AZGP1 promotes metastasis, AZGP1 interactors were purified using immunoaffinity purification and resolved using liquid chromatography and high-throughput mass spectrometry (LC-MS/MS) (Fig. 3A). 75 Possible binding proteins were obtained. 8 of them were selected for further verification by searching at the UniProt database (http://www.uniprot.org) and literature reviews (Fig. 3B). We found actin-binding protein filamin A (FLNA) may be the most potential interactor with AZGP1. The interactions between both exogenous and endogenous FLNA with AZGP1 were confirmed by co-immunoprecipitation (co-IP) in HEK293, HCT116, and SW480 cells (Fig. 3C-D). Numerous studies show the activation of FLNA is directly related with oncogenesis and metastasis in different cancers, we assumed that AZGP1 promoted cancer development and metastasis mediated by FLNA.

In order to verify this conjecture, rescue experiments were conducted. Firstly, lentivirus mediated FLNA shRNA were constructed and the protein expression were verified by Western Blot after infected. Then, transwell assays indicated that over expressed AZGP1 promoted migration, while the migration promotion was damaged when FLNA was down regulated (Fig. 3E). Besides that, the epithelial-to-mesenchymal transition showed the same result. When AZGP1 was over expressed, it showed upregulation of N-cadherin and Vimentin. However, these phenomena reversed if FLNA was downregulated (Fig. 3F).

Collectively, these data provide biochemical evidence that AZGP1 interacts with FLNA and facilitates CRC cell metastasis mediated by FLNA.

### PAK2 mediated phosphorylation of FLNA involved in the promotion metastasis by AZGP1

**Figure 4:**
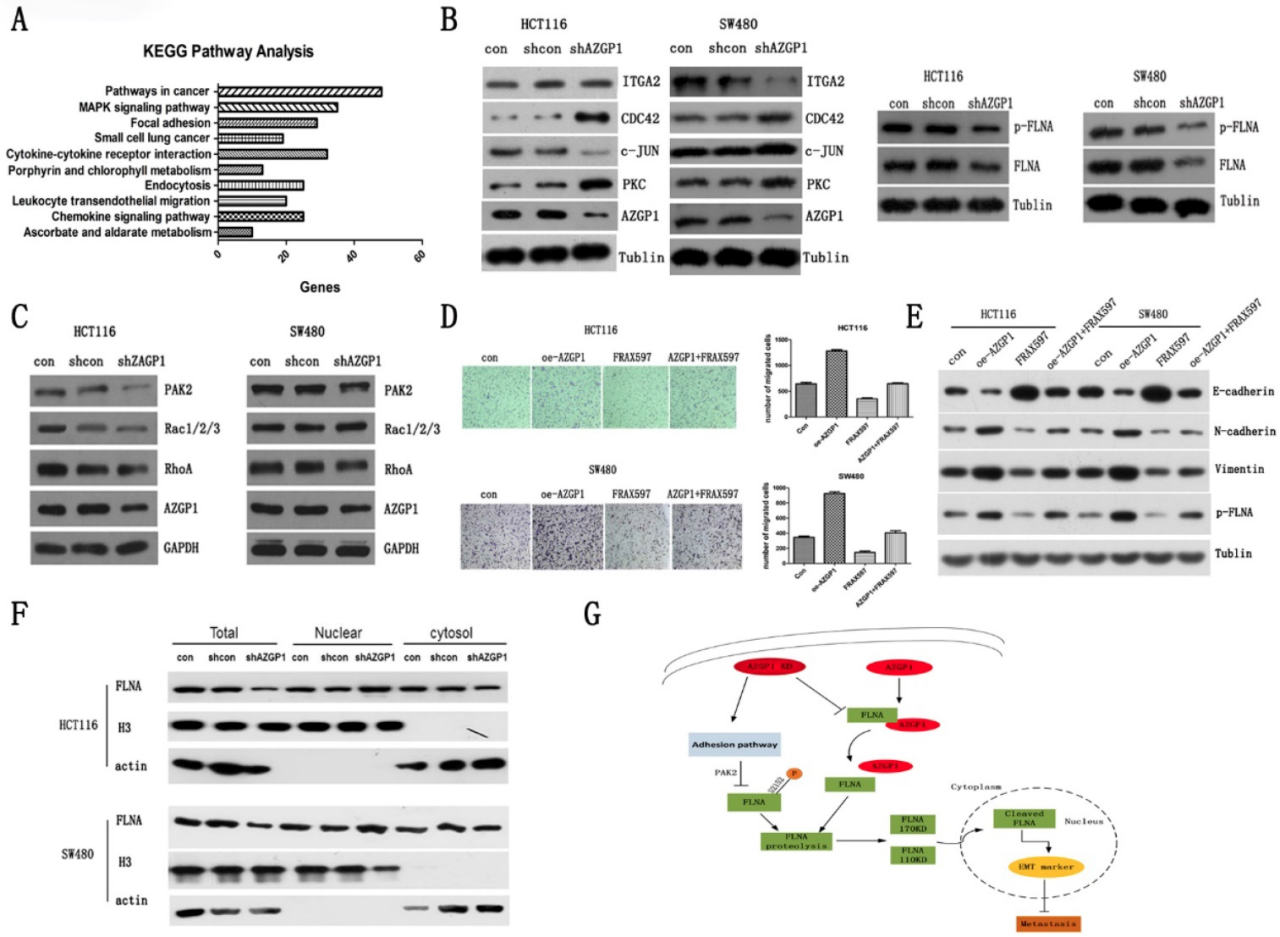
** PAK2 mediated phosphorylation of FLNA involved in the promotion metastasis by AZGP1.** KEGG pathway analysis from microassay. (B) Western blot analysis of proteins involved in the adhesion pathway. And the general expression and phosphorylation of FLNA when AZGP1 was down-regulated. (C)Western blot analysis of proteins PAK2, RhoA, Rac1/2/3. (D) Transwell assay the migration after over-expressed AZGP1 and treated with PAK2 inhibitor FRAX597. (E)Western blot analysis the marker of EMT and phosphorylation of FLNA after over-expressed AZGP1 and treated with PAK2 inhibitor FRAX597 (1uM, 48h). (F)Distribution of FLNA, a conductor protein of AZGP1, in the cytoplasm and nucleus.(G) Proposed mechanism by which AZGP1 promotes metastasis: when AZGP1 is highly expressed, it is inclined to interact with FLNA, preventing its proteolysis. When knock down, it prevents the interaction. Besides, it regulated by adhesion pathway and inhibited phosphorylation of FLNA by restrained PAK2, then, facilitated its proteolysis and its distribution to the nucleus, thereby suppression metastasis.

To determine the specific pathway by which AZGP1 promotes metastasis, the gene expression profile was first examined using an Affymetrix microassay following knockdown of AZGP1. Among the genes identified, 801 were upregulated, while 591 were downregulated. KEGG pathway analysis was performed, and it suggested that the focal adhesion pathway is responsible for the regulation of metastasis (Fig. 4A). The corresponding changes in proteins, such as PKC, CDC24, ITGA2, and c-JUN supported this conclusion. Additionally, the AZGP1-binding protein, FLNA, another focal adhesion protein, decreased after silencing AZGP1, as well as the phosphorylation of FLNA (Fig. 4B). Numerous studies have shown FLNA is directly related to the EMT of tumor cells, mostly, the phosphorylation of FLNA determined its molecular biology function. Hence, we try to find out the possible kinases regulating phosphorylation of FLNA.

PAK2, one member of p21-activating kinases (PAKs), mediates oncogenic effects by phosphorylating multiple substrates involved in proliferation, migration and cytoskeletal reorganization. As shown in Figure 4C, the expression of PAK2 declined when silencing AZGP1. Additionally, it was one of the downregulated genes in microassay and verified by qPCR (data was not shown). Rescue experiment showed that over-expressed AZGP1 promoted the migration of colorectal cancer, but it can be reversed when the PAK2 inhibitors are added (Fig. 4D). Similarly, Western Blot showed that over-expressed AZGP1 promoted EMT and phosphorylation of FLNA, and PAK2 inhibitors can reverse this change (Fig. 4E). In the current study, the repression of FLNA especially its phosphorylation was due to the inactivation of PAK2 when knock-down of AZGP1 occurred.

Multiple studies have shown that FLNA exhibited opposing roles in cancer progression depending on its location. In prostate cancer, the cytoplasmic localization of FLNA determines a propensity to metastasize that can be prevented by cleavage and subsequent nuclear translocation 24. We also found a reduction in total FLNA but increased recruitment to the nucleus (Fig. 4F). Besides, the phosphorylated FLNA did not differ significantly compared to FLNA knock-down of AZGP1, these results indicate that the interaction with AZGP1 is required to prevent FLNA proteolysis, once the interaction is broken, the fragment may prefer to reposition to the nucleus, where it suppresses metastasis. And, silencing AZGP1 also prevented the activity of serine-threonine kinase PAK2 and inhibited the phosphorylation of FLNA at S2152, accordingly, enhanced the instability of FLNA (Fig. 4G).

## Discussion

Liver metastasis is the main cause of CRC mortality, however, the mechanism remains largely unknown. To identify the genes responsible for liver metastasis, we used a microassay to compare primary tumors without liver metastasis to those with liver metastasis. AZGP1 was identified as a promising molecular regulating liver metastasis. In this study, we demonstrated that AZGP1 is highly expressed in CRC tissues with liver metastasis. Moreover, abundant expression of AZGP1 was associated with a poor prognosis. Additional molecular biology experiments demonstrated that AZGP1 promotes metastasis by mechanistically interacting with FLNA and regulating the adhesion pathway.

Recent studies have shown that AZGP1 regulates a variety of tumors. AZGP1 expression has, in general, been reported as downregulated in certain carcinomas, including those in the breast, prostate, liver, and stomach. However, increased expression has been reported in other tumors, such as lung adenocarcinoma. Moreover, AZGP1 was significantly upregulated in a tissue microarray containing 190 samples of primary colon cancer tissue compared with normal colonic tissue. On this basis, we found that AZGP1 is highly expressed in CRC tissue with liver metastasis relative to those without liver metastasis. Logistic regression analysis also revealed that abundant expression of AZGP1 is a risk factor for metastasis in CRC.

Currently, abnormal expression of AZGP1 can be used as an important indicator for prognosis, however, the mechanism in cancer progression remains largely unknown. In hepatocellular carcinoma, the underlying molecular mechanism by which AZGP1 inhibits cell migration and invasion is regulating the PTEN/Akt and CD44 pathways 25. Additionally, previous reports have demonstrated that AZGP1 suppresses the EMT or induces mesenchymal-to-epithelial trans-differentiation by blocking the TGF-β-ERK pathway in hepatocellular carcinoma and pancreatic cancer 20, 21. Considered as an adipokine, some researchers have indicated that AZGP1 may regulate the malignant phenotype by energy metabolism and the mTOR signaling pathway 26, 27. In our study, we demonstrated that AZGP1 regulated CRC cell metastasis by interacting with FLNA and regulating the focal adhesion pathway.

FLNA, an actin-binding protein, is important for organogenesis during development. Previous reports have revealed its involvement in cancer development 28. As a large molecule (280-kDa), FLNA is highly susceptible to proteolysis or cleavage 29, 30. Multiple studies have shown that full-length FLNA is mainly localized to the cytoplasm and localizes to the nucleus once it is cleaved to its 90-kDa fragment. Consequently, it can cause two opposite outcomes depending on its subcellular localization. The presence of full-length FLNA in the cytoplasm promotes cell growth and metastasis 31 but inhibits cell growth and prevents trans-activation, forming part of the pre-initiation complex in the nucleus 32, 33. The susceptibility of FLNA to cleavage is regulated by its phosphorylation; it can be phosphorylated at S2152 by several different kinases, including cyclin-dependent kinase 1 (Cdk1), protein kinase A (cAMP-dependent protein kinase), protein kinase C (PKC), and Ca2+/calmodulin-dependent protein kinase II 34-37. Researchers have shown that PKC is the common phosphokinase. Stimulation of PKC activity in cells usually results in FLNA phosphorylation. But in our study, when silencing AZGP1, we detected upregulated protein expression and kinase activity of PKC (data was not shown), while the specific phosphorylation at S2152 of FLNA did not increase consistently compared with p-FLNA and FLNA. Fortunately, another phosphokinase PAK2 decreased which was consistent with down-regulation of FLNA. FLNA undergoes proteolysis to prevent further activation of its downstream targets, such as EMT markers, to prevent unwanted cell migration. Overall, FLNA is used as a scaffold for other proteins to bind to and regulate the response to cell signaling. FLNA has more than 90 known protein-binding partners. Additionally, most protein binding occurs at its C-terminal end 38. In this study, AZGP1 was identified as a novel FLNA binding partner that promotes metastasis in CRC cells. However, the exact binding site should be explored in more depth.

In summary, our results show that AZGP1 is an oncogene that plays an important role in promoting liver metastasis in CRC. AZGP1 knockdown prevented cancer cell EMT, which could contribute to the inhibition of metastasis. The mechanism involved is correlated with the activation of the focal adhesion pathway for example increased expression of CDC24. In addition, FLNA phosphorylation inhibition occurred due to the reduced PAK2 activity and then contributed to its instability. The dissociation of FLNA and its subsequent proteolysis promoted its nuclear localization accordingly. Finally it regulated the EMT marker and prevented the formation of distant metastases. Thus, silencing AZGP1 could be an efficient method to prevent metastasis and improve OS in CRC patients by delaying tumor progression.

## Figures and Tables

**Table 1:** Relationships between AZGP1 expression in tumor tissues and patient/tumor clinic-pathological characteristics.

	High (%) n=106	Low (%) n=174	Correlation coefficient	P value
Sex			0.036	0.552
Male	62(58.5)	108(62.1)		
Female	44(41.5)	66(37.9)		
Age - years			-0.051	0.335
≤60	47(44.3)	67(38.5)		
>60	59(55.7)	107(61.5)		
Primary tumor site			-0.079	0.414
Right-sided	39(36.8)	55(31.6)		
Left-sided	29(27.4)	41(23.6)		
Rectum	38(35.8)	78(44.8)		
Primary tumor size -cm			-0.127	0.104
<3	37(34.9)	53(30.5)		
3-5	51(48.1)	72(41.4)		
≥5	18(17.0)	49(28.2)		
Primary histological type			-0.009	**0.029**
Non-mucinous	88(83.0)	150(86.2)		
Mucinous	18(17.0)	17(9.8)		
Others	0(0)	7(4)		
Primary differentiation			0.033	0.582
Well to moderate	66(62.3)	114(65.5)		
Poor	40(37.7)	60(34.5)		
Primary pT stage			0.067	0.263
1/2	13(12.3)	30(17.2)		
3/4	93(87.7)	144(82.8)		
Primary pN stage			0.063	0.153
No	51(48.1)	99(56.9)		
Yes	55(51.9)	75(43.1)		
Vascular invasion			-0.034	0.570
No	101(95.3)	163(93.7)		
Yes	5(4.7)	11(6.3)		
Nerve invasion			0.062	0.301
No	104(98.1)	173(99.4)		
Yes	2(1.9)	1(0.6)		
Clinical stage			0.112	**0.003**
I-III	57(53.8)	124(71.3)		
IV	49(46.2)	50(28.7)		

Correlation coeffcient and P value: Cramer's V in Pearson's χ^2^ test The pathological tumor stage was documented according to the 7^th^ AJCC TNM classification.
