# Zinc-α2-glycoprotein 1 promotes EMT in colorectal cancer by filamin A mediated focal adhesion pathway: Erratum

**DOI:** 10.7150/jca.87171

**Published:** 2023-07-26

**Authors:** Meiling Ji, Wenxiang Li, Guodong He, Dexiang Zhu, Shixu Lv, Wentao Tang, Mi Jian, Peng Zheng, Liangliang Yang, Zhipeng Qi, Yihao Mao, Li Ren, Yunshi Zhong, Yongjiu Tu, Ye Wei, Jianmin Xu

**Affiliations:** 1 Department of General Surgery, Zhongshan Hospital Fudan University, Shanghai, China; 2 Department of Surgical Oncology, First Affiliated Hospital of Wenzhou Medical University, Wenzhou, China; 3 Departmentof Endoscopic Center, Zhongshan Hospital Fudan University, Shanghai, China; 4 Surgical Department, Hospital 174 of PLA, Xiamen, Fujian, China

In the initially published article, we recently realized that there are some mistakes in figures. It occurred while typesetting, but these results do not affect our main conclusion of the study. The corrections are provided below.

## Figures and Tables

**Figure 2:**
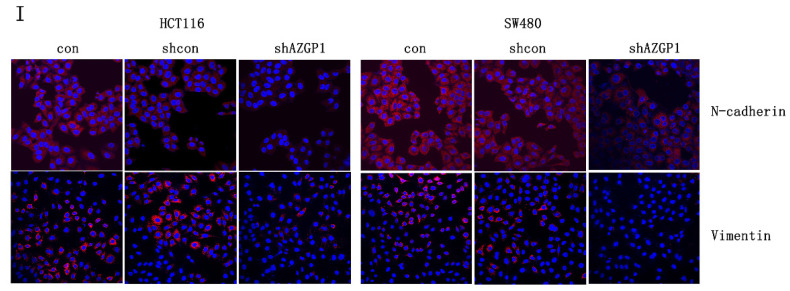
Corrected Figure 2I. (I) Representative immunofluorescence images of EMT markers N-cadherin and Vimentin.

**Figure 3:**
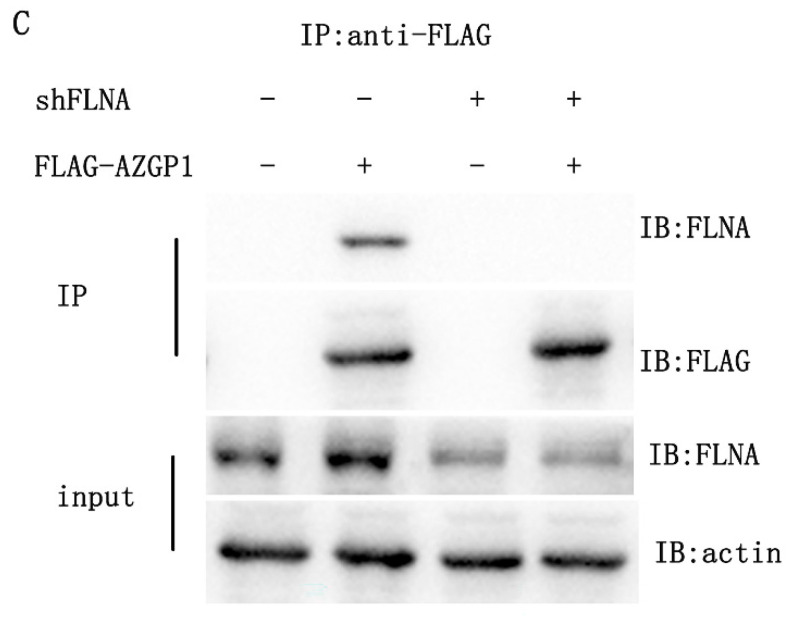

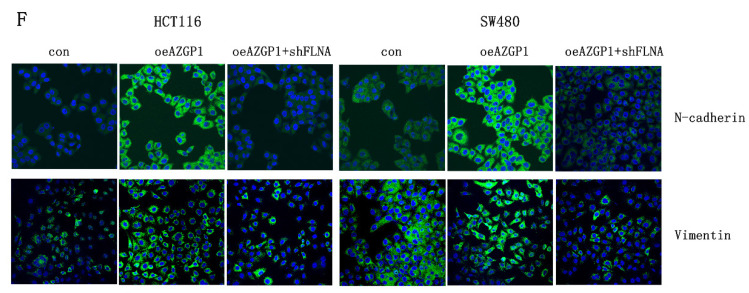
Corrected Figure 3C and 3F. (C) Co-immunoprecipitation of AZGP1 and FLNA was conducted in 293 cells. (F) Immunofluorescence staining of EMT markers in overexpressing AZGP1 (oeAZGP1) and knocking down FLNA.

